# HSP90: A Novel Target Gene of miRNA-628-3p in A549 Cells

**DOI:** 10.1155/2018/4149707

**Published:** 2018-05-20

**Authors:** Jieli Pan, Fusheng Jiang, Jia Zhou, Dehong Wu, Zhenhua Sheng, Meiya Li

**Affiliations:** ^1^Academy of Chinese Medical Sciences, Zhejiang Chinese Medical University, Hangzhou 310053, China; ^2^College of Life Science, Zhejiang Chinese Medical University, Hangzhou 310053, China; ^3^College of Basic Medical Sciences, Zhejiang Chinese Medical University, Hangzhou 310053, China; ^4^The Second Affiliated Hospital of Zhejiang Chinese Medical University, Hangzhou 310053, China

## Abstract

Lung cancer is one of the leading causes of cancer-related death in the world. MicroRNA- (miR-) 628-3p plays critical roles in many cancers, including lung cancer. We investigated how miR-628-3p affected migration and apoptosis in A549 cells. We used bioinformatics algorithms to predict the miR-628-3p target gene to study the molecular mechanism by which miR-628-3p contributes to lung cancer. Then, we used the luciferase reporter assay to identify whether heat shock protein 90a* (HSP90)* is a direct target of miR-628-3p. Western blotting and quantitative real-time PCR showed that miR-628-3p downregulated HSP90a protein expression via a posttranscriptional mechanism. We confirm that miR-628-3p promotes apoptosis and inhibits migration in A549 cells by negatively regulating* HSP90*. Our results may reveal a novel strategy for lung cancer treatment.

## 1. Introduction

Lung cancer is the most common cancer in the world and is the leading cause of cancer-related deaths [1]. The occurrence and development of lung cancer are a complex, multistep, and multilevel process involving multiple factors, and the mechanism involved remains unknown. Lung cancer has become a challenging topic and hotspot in tumor research. Although research on the early diagnosis and treatment of lung cancer in recent years has become more in depth, the overall prognosis and survival of patients with lung cancer are unsatisfactory; therefore, there is still much to be done in lung cancer research and treatment.

There are many types of lung cancer, among which non-small cell lung cancer (NSCLC) is the main type, accounting for about 75–80% of lung cancer cases [2]. As the symptoms are not apparent and detection is difficult at stage I and II NSCLC, it is mostly diagnosed at the late stages, which reduces the five-year survival rate. Therefore, earlier detection of lung cancer is needed and could lead to more effective management of the disease.

There are a variety of abnormal factors in lung cancer [3, 4]; research into the mechanism of action has been performed at molecular level, but the exact molecular mechanism remains unclear. With the development of molecular biology technology, the discovery of microRNAs (miRNAs) has opened up a new avenue in lung cancer research and provides new ideas and approaches for diagnosing and treating lung cancer.

miRNAs are a family of naturally occurring, small, endogenous noncoding RNAs that are 18–25 nucleotides in length [5]. miRNAs influence many biological processes, including cell proliferation, apoptosis, development, differentiation, migration, and survival. miRNAs are differentially expressed in cancer and play important roles in regulating gene expression by base-pairing to the complementary sites on the target mRNAs, consequently blocking translation or triggering the degradation of the target mRNAs [6, 7]. Growing evidence has demonstrated the role of miRNAs in many human tumors, including breast [8], ovarian [9], pancreatic [10], thyroid [11], and lung cancers [1].

The altered expression of tissue miRNAs has been associated with many diseases, particularly cancer. In recent years, the role of miR-628-3p in cancer has received increased attention. miR-628-3p is abnormally expressed in many cancers, such as colon [12], gastric [13], and pancreatic cancer [14]. The most recent study, which performed TaqMan low-density DNA microarray comparative analysis of more than 200 cases of early lung cancer and more than 170 cases of normal human plasma samples, found abnormal high expression miR-628-3p in the lung cancer cases, suggesting that miR-628-3p can be used as a marker of early diagnosis of lung cancer [15]. However, the regulatory mechanism of miRNA in lung cancer is not clear, and more research is needed.

At present, numerous clinical studies have confirmed that the remission rate of molecular targeted drugs in patients with lung cancer is not very high, and drug resistance against targeted drugs is increasing. Therefore, further study of the main target in lung cancer is imperative, and developing lung cancer treatment closely related to miRNA-target research has gradually become a hot research topic. Identifying the target of miR-628-3p and clarifying the mechanism of miR-628-3p would provide new ideas and methods for diagnosing and treating lung cancer.

Heat shock protein 90a (HSP90a), one of the most abundant and conserved chaperone proteins, aids in preserving the integrity and function of numerous client proteins. Plasma HSP90a protein levels are useful as a diagnostic biomarker in lung cancer [16].

In this study, we investigated the relationship between miR-628-3p and HSP90a in A549 lung cancer cells. Moreover, we provide evidence that* HSP90* is a target gene of miR-628-3p in A549 cells.

## 2. Materials and Methods

### 2.1. Cell Culture

293T and A549 cells were cultured in Dulbecco's modified Eagle's medium (Invitrogen, USA) supplemented with 10% (v/v) fetal bovine serum (Gibco, USA) and 1% antibiotics (100 U/mL penicillin and 100 mg/L streptomycin sulfate). All cells were grown in a humidified atmosphere at 37°C with 5% CO_2_.

### 2.2. Oligonucleotide Transfection

miR-628-3p mimics and* HSP90* small interfering RNAs (siRNAs) were designed and synthesized by RiboBio (Guangzhou, China); Table 1 lists their sequences and the targeted mRNAs.

Cells were plated on 6-well plates (Corning, USA) and cultured overnight to 40–60% confluence. Transfection was performed with Lipofectamine 3000 (Invitrogen, USA) according to the manufacturer's protocol. After 48 h, we harvested the cells and detected HSP90 and miR-628-3p by western blotting and quantitative real-time polymerase chain reaction (qRT-PCR), respectively.

### 2.3. Apoptosis Assay

Apoptosis was determined by annexin V and propidium iodide (PI) staining using an apoptosis detection kit (556547, BD) according to the manufacturer's instructions. Briefly, A549 cells were transfected with 50 nM NC mimic, miR-628-3p mimic, or* HSP90* siRNAs for 48 h. Then, the cells were washed with 50 mmol/L cold phosphate buffer (pH 7.5), centrifuged at 1200 ×g for 5 min, and suspended in binding buffer.

The treated cells were incubated with annexin V and PI for 15 min at room temperature and then analyzed for annexin V binding affinity within 1 h by flow cytometry (BD Accuri C6).

### 2.4. RNA Extraction and qRT-PCR

Total RNA from the cells was extracted using TRIzol (Invitrogen) according to the manufacturer's protocol. Complementary DNA (cDNA) was synthesized from 1 *μ*g total RNA with a PrimeScript reverse transcription (RT) reagent kit with genomic DNA (gDNA) Eraser (TaKaRa, China) in a 20 *μ*L volume [mRNA genes, RT primers; miRNA, U6 rRNA, and miRNA-specific primers (Bulge-Loop miRNA quantitative PCR (qPCR) primers, RiboBio)]. Real-time PCR was carried out with SYBR Green I mix reagents (TaKaRa, Dalian, China) in a 20 *μ*L reaction volume (10 *μ*L SYBR Green I mix, 200 mM forward and reverse primer, 1 *μ*L cDNA template) on a 7500 Real-Time PCR System (Applied Biosystems). Each reaction was run in triplicate; the threshold cycle (Ct) data were determined by fixed threshold settings, and the average Ct of triplicate PCR was calculated. The relative miRNA levels were confirmed using the comparative Ct method. The amount of internal control U6-related miRNA was calculated using the equation 2^−ΔΔCt^, in which ΔΔCt = (Ct miRNA − Ct U6) tumor − (Ct miRNA − Ct U6) control. The relative amount of* HSP90* mRNA was normalized to 18s rRNA using a method similar to the one described above.

### 2.5. Protein Extraction

Cells were washed three times with phosphate-buffered saline (PBS) chilled to 4°C. Whole-cell proteins were extracted with M-PER Mammalian Protein Extraction Reagent (78503, Thermo Fisher Scientific, USA) containing protease and phosphatase inhibitor (Roche, Germany) at 4°C for 30 min. Then, the samples were centrifuged at 14,000 ×g for 10 min, and the supernatant was transferred to a new tube for analysis.

### 2.6. Automated Western Immunoblotting

Before blotting, the protein was quantified using the bicinchoninic acid (BCA) method. Simple western immunoblotting was performed on a Peggy Sue system (ProteinSimple, California, USA) using a Size Separation Master Kit with Split Buffer (12–230 kDa) according to the manufacturer's standard instruction and using anti-HSP90 (4877S, CST, USA) and anti-*β*-actin (4970S, CST, USA) antibodies. Compass software (version 2.7.1, ProteinSimple) was used to program the Peggy Sue and for presentation (and quantification) of the western immunoblots. Output data were displayed from the software-calculated average of seven exposures (5–480 s).

### 2.7. Luciferase Reporter Assay

We used luciferase reporter assays to verify whether miR-628-3p directly targets the* HSP90* gene and used a pHY-REPORT system (Hanyin Biotechnology, Shanghai, China). Briefly, 46-mer double-stranded oligonucleotides containing the predicted miRNA binding sites in* HSP90* were synthesized and ligated between the* Xho*I and* Bam*HI restriction sites of the pHY-REPORT luciferase vector to establish the pLUC-HSP90 vector.

A549 cells were cotransfected with 50 nM miR-628-3p mimic or negative control (NC) mimic in 96-well plates containing 200 ng/*μ*L pLUC-HSP90 or pLUC-mutHSP90 plasmid and 10 ng* Renilla* luciferase (internal transfection efficiency control). After 24-h transfection, luciferase activity was detected using a Dual-Luciferase Reporter Assay System (Promega, USA) according to the manufacturer's protocol. Luminescence intensity was read with a microplate luminometer using the corresponding Promega protocol. Transfections were performed in duplicate and repeated three times.

### 2.8. Wound Healing Assay

An* in vitro* wound healing assay was performed to measure the unidirectional migration of A549 cells. A549 cells (10 × 10^4^ cells/mL) were seeded in 6-well plates and were allowed to grow for 24 h after being transfected with miR-628-3p mimic, NC mimic, or* HSP90* siRNAs. Then, the A549 monolayers were scratch-wounded in a straight line using a 10 *μ*L pipette tip when cells were 70–80% confluent. Immediately after wounding and after 24 h incubation, the cells were photographed under a microscope (Axio Observer A1, Zeiss, Germany). Migration was calculated as the area of A549 cells that had migrated from the injured edge in to the wound zone. At least four points in each of three random fields were examined for each of three independent wounds. The wound closure (%) was calculated as migrated cell surface area/total surface area × 100.

### 2.9. Statistical Analysis

All data are presented as the mean ± SEM and were analyzed using SPSS16 software. *p* values were calculated using Student's unpaired* t*-test (for two groups) or *Z*-way analysis of variance (for more than two groups). Statistical significance was taken at *p* < 0.05 and *p* < 0.001.

## 3. Results

### 3.1. Screening of miR-628-3p Target Genes

TargetScan (http://www.targetscan.org/mamm_31/) and NCBI (https://www.ncbi.nlm.nih.gov/) were used to analyze the information of multiple genes that can bind with miR-628-3p, and we selected nine genes with a high degree of integration:* FAS*,* APAF1*,* XIAP*,* HSP90*,* PIK3R3*,* RIPK1*,* CASP3*,* PMAIP1*, and* AKT2*.

**Figure 1:**
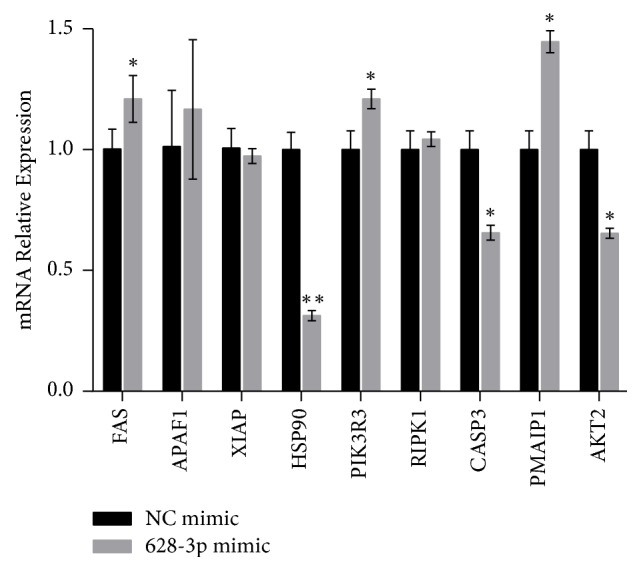
*miR-628-3p and target gene expression in 293T cells*. ^*∗*^*p* < 0.05, ^*∗∗*^*p* < 0.001.

To further screen target genes, miR-628-3p mimic was transfected into 293T cells, and quantitative RT-PCR (qRT-PCR) was used to detect the expression levels of the predicted genes as regulated by miR-628-3p.  Figure 1 shows that* FAS*,* PIK3R3*, and* PMAIP1* were upregulated after miR-628-3p mimic transfection, while* HSP90*,* CASP3*, and* AKT1* were downregulated; among the three downregulated genes,* HSP90* was significantly downregulated, so it was selected for further research.

### 3.2. miR-628-3p Was Expressed High in A549 Cells

**Figure 2:**
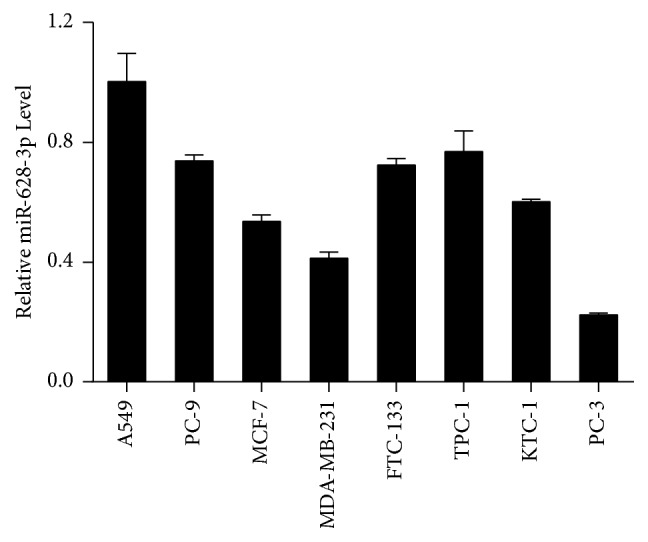
*miR-628-3p is expressed differentially in different cancer cells*. Relative levels of miR-628-3p in A549 cells were normalized against that of the other cell lines. *n* = 5, mean ± SD.

We used eight cancer cell lines (A549, PC-9, MCF-7, MDA-MB-231, FTC-133, TPC-1, KTC-1, and PC-3) to select a cell line in which to research the regulatory relationship between* HSP90* and miR-628-3p. miR-628-3p expression levels in the cell lines were compared using qRT-PCR. As miR-628-3p expression was higher in A549 cells than in the other cancer cells (Figure 2), we selected the A549 cell line for further research.

### 3.3. The Expression of miR-628-3p Was Efficient after Transfection in A549 Cells

**Figure 3:**
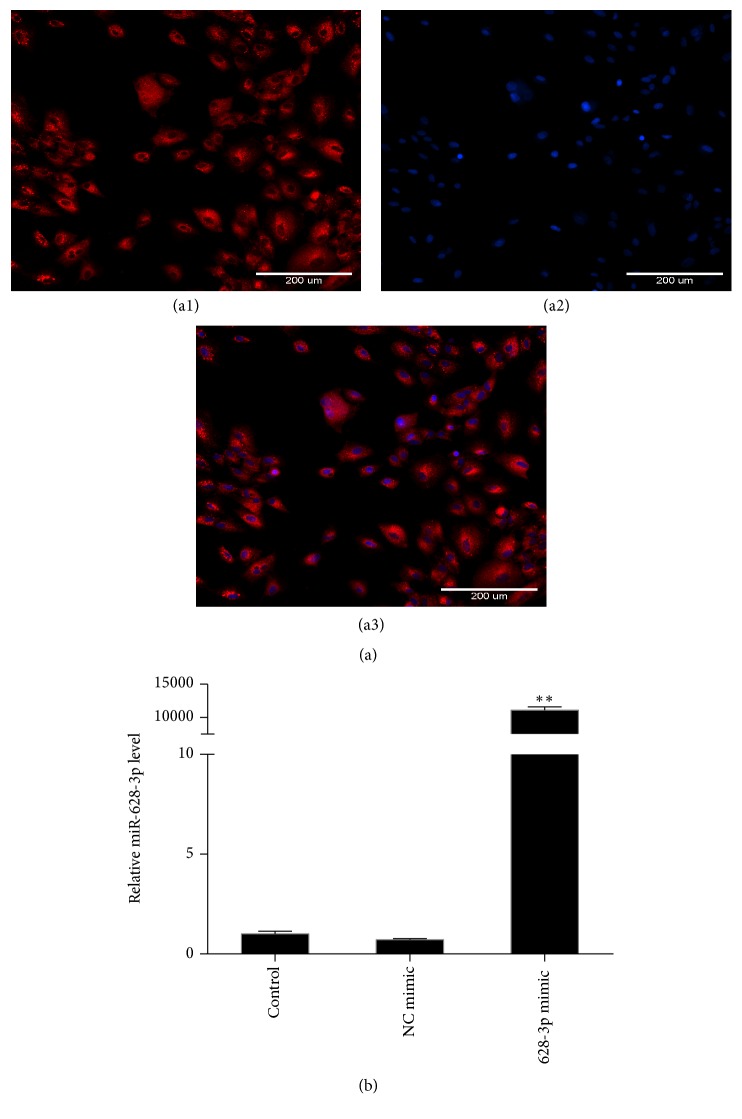
(a) Immunofluorescence images of A549 cells transfected with miR-628-3p. (b) Relative miR-628-3p expression levels after transfection in A549 cells. ^*∗∗*^*p* < 0.001.

Cy3 was used as an indicator to verify the vector transfection efficiency. After 24-h transfection, the cells were photographed by fluorescence microscopy, and each cell emitted red fluorescence (Figure 3(a)), indicating that vector transfection efficiency was very high. To verify miR-628-3p expression, A549 cells were transfected with miR-628-3p mimic or NC mimic (vector control); the cells that were not transfected with vector were used as the blank control. miR-628-3p expression was detected by fluorescence qPCR (Figure 3(b)), which showed that cells transfected with miR-628-3p mimic had far higher miR-628-3p expression than the control, indicating that miR-628-3p mimic expression was very efficient.

### 3.4. HSP90 Protein Was Downregulated in A549 Cells Transfected with miR-628-3p Mimic

**Figure 4:**
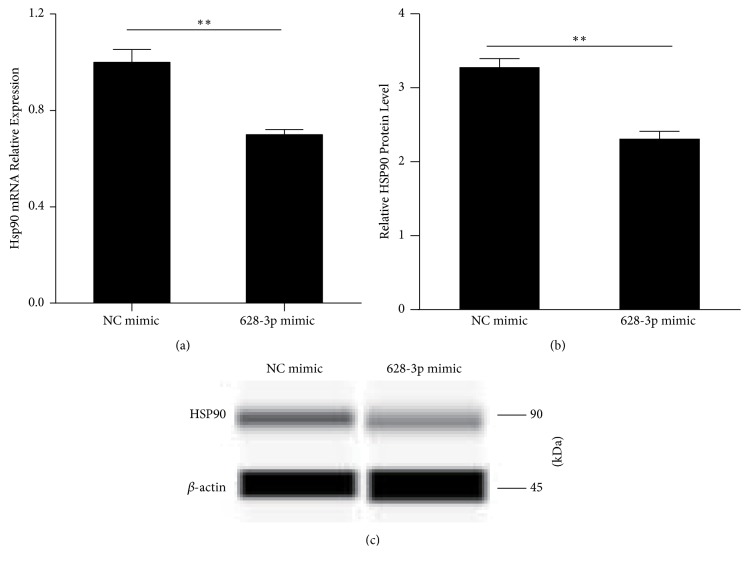
(a)* HSP90* mRNA was downregulated in A549 cells transfected with miR-628-3p mimic. The average mRNA expression from the NC group is designated as 1. (b, c) Western blot measurement of HSP90 protein levels after 48-h transfection with miR-628-3p mimic. ^*∗∗*^*p* < 0.001.

Western blotting was used to analyze the regulatory relationship between miR-628-3p and HSP90 in A549 cells following transfection with miR-628-3p mimic or NC mimic. Immunoblotting with antibodies against HSP90 and *β*-actin showed that miR-628-3p obviously inhibited HSP90 expression (Figure 4), indicating a negative regulatory relationship between miR-628-3p and HSP90.

### 3.5. miR-628-3p Regulated HSP90 Expression by Targeting Its 3′ UTR

**Figure 5:**
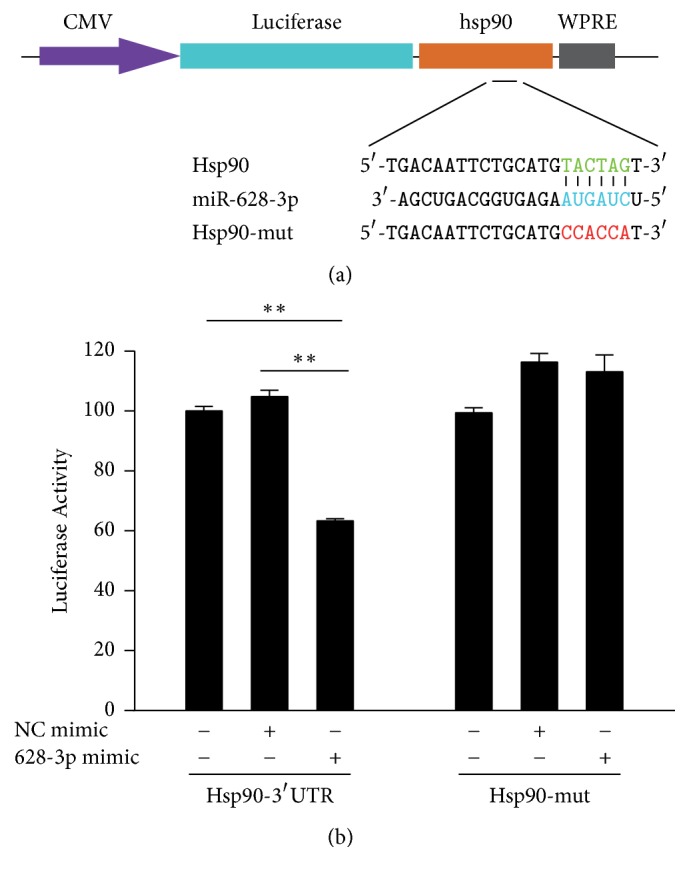
*miR-628-3p directly regulates HSP90 expression in A549 cells by targeting the HSP90 3*′* UTR*. (a) Predicted consequential pairing of* HSP90* 3′ UTR and miR-628-3p by TargetScan. (b) Luciferase assay in A549 cells. pLUC-HSP90 vector was cotransfected with miR-628-3p mimic or NC mimic. Luciferase activity in pLUC-HSP90 A549 cells was significantly decreased following ectopic expression of miR-628-3p mimic. ^*∗∗*^*p* < 0.001.

Luciferase assay was used to confirm whether the 3′ untranslated region (3′ UTR) of the predicted target genes had putative miR-628-3p-binding sites.* HSP90* was selected as a potential target gene of miR-628-3p. TargetScan predicted the consequential pairing of the* HSP90* 3′ UTR and miR-628-3p (Figure 5(a)), where the dual-luciferase reporter assay showed that miR-628-3p directly bound to the* HSP90* 3′ UTR in A549 cells. To test the* HSP90* putative binding site, we generated a luciferase reporter (pLUC-HSP90) and a mutant HSP90 construct (pLUC-mutHSP90) in which the* HSP90* 3′ UTR was altered using a site-directed mutagenesis kit. miR-628-3p significantly regulated the luciferase activity of the HSP90 construct (Figure 5(b)), whereas the mutant construct did not generate luciferase activity. These results indicate that miR-628-3p may regulate* HSP90* expression by targeting its 3′ UTR.

### 3.6. siRNA Transfection Interfered with Downregulated HSP90 Expression

**Figure 6:**
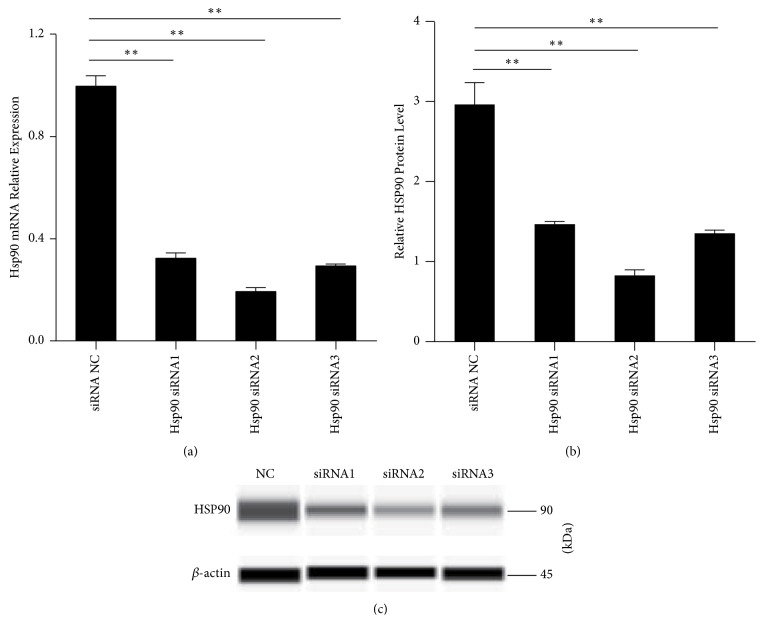
(a) qRT-PCR measurement of* HSP90* relative expression after 48-h siRNA transfection.* HSP90* mRNA was downregulated in the siRNA-transfected A549 cells. The average* MIR628-3P* mRNA expression from the NC group is designated as 1. (b, c) Western blot measurement of HSP90 protein after 48-h transfection. HSP90 protein was downregulated in the siRNA-transfected A549 cells. ^*∗∗*^*p* < 0.001.

Three siRNAs targeting the* HSP90* gene were transfected into the A549 cells, and the relative mRNA expression was measured 48 h after transfection. siRNA2 is better than the other two siRNAs (Figure 6(a)). HSP90 protein expression was detected by western blotting; siRNA2 interference downregulated HSP90 expression significantly as compared to the other two siRNAs (Figures 6(b) and 6(c)) significantly.

### 3.7. miR-628-3p Induced Apoptosis in A549 Cell

**Figure 7:**
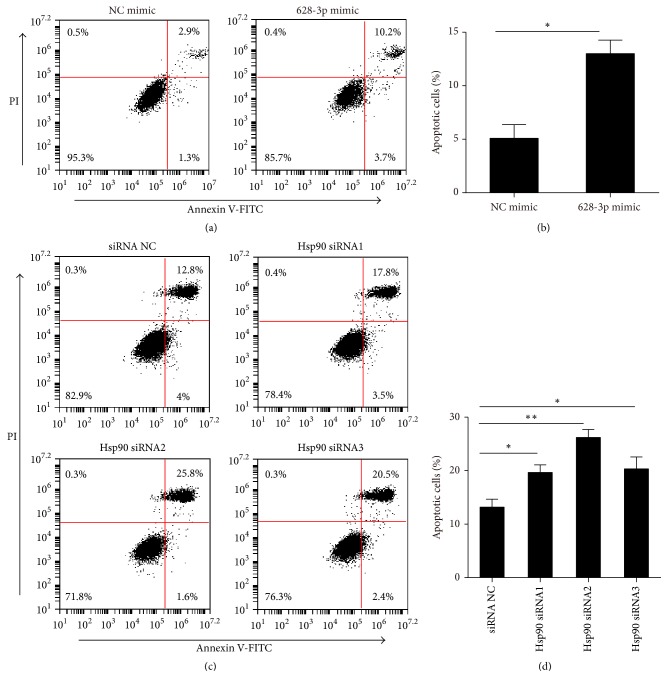
*HSP90 expression levels affect A549 cell apoptosis*. (a, b) miR-628-3p mimic transfection induced apoptosis in A549 cells. (c, d)* HSP90* siRNA transfection induced apoptosis in A549 cells. ^*∗*^*p* < 0.05, ^*∗∗*^*p* < 0.001.

The rate of apoptosis in the A549 cells was measured by flow cytometry with annexin V and PI staining following 48-h transfection. Figures 7(a) and 7(b) show the percentage of early or late apoptotic cells following transfection with NC mimic and miR-628-3p mimic, and Figures 7(c) and 7(d) show the percentage of early or late apoptotic cells following transfection with the control and the three* HSP90* siRNAs. Transfection with miR-628-3p mimic and the* HSP90 *siRNAs induced apoptosis in the A549 cells.

### 3.8. miR-628-3p Inhibited Migration in A549 Cells

**Figure 8:**
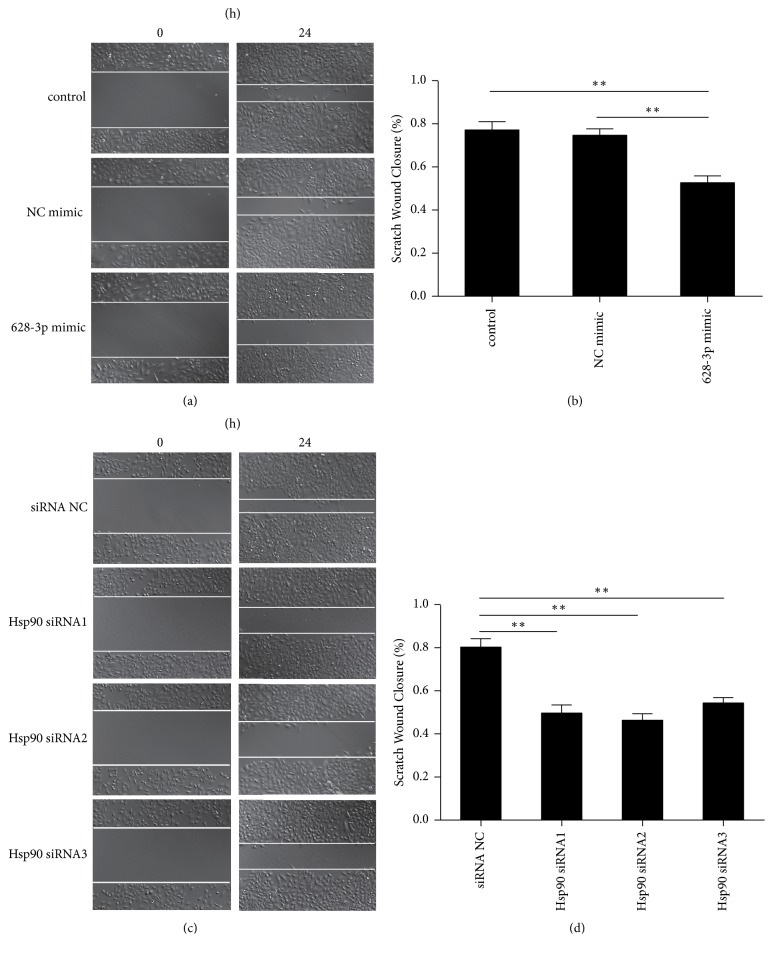
*RNA interference of HSP90 inhibits wound healing*. (a) A549 cells transfected with control, NC mimic, and miR-628-3p mimic photographed at 0 h and 24 h after wounding. (b) Scratch wound closure in A549 cells transfected with control, NC mimic, and miR-628-3p mimic. (c) A549 cells transfected with NC or HSP90 siRNAs photographed at 0 h and 24 h after wounding. (d) Scratch wound closure in A549 cells transfected with NC or HSP90 siRNAs. ^*∗∗*^*p* < 0.001.

To understand how miR-628-3p affects cell migration, A549 cells were transfected with* HSP90* siRNAs or miR-628-3p mimic. Transfection with miR-628-3p mimic inhibited A549 cell migration (Figures 8(a) and 8(b)), as did transfection with the* HSP90* siRNAs (Figures 8(c) and 8(d)), suggesting that miR-628-3p overexpression and interference with HSP90 expression have the same effect of decreasing A549 cell migration.

## 4. Discussion

The 2018 US cancer statistics reported an estimated 1,735,350 new cases and 609,640 new deaths from lung and branches cancer in the United States, indicating that lung and branches cancer is the leading cause of death among all tumors in the world [17]. Although the development of diagnostic and prognostic techniques has improved the survival of patients with lung cancer, most patients are diagnosed at the late stage. miRNAs function as tumor suppressors or oncogenes in various human cancers, including lung cancer [18]. Therefore, identifying the tumor-associated miRNAs and their target genes that underlie lung carcinogenesis might reveal novel therapeutic targets.

Some studies have indicated that miRNAs could play an important role in the initiation and progression of lung cancer; for example, overexpression of miR-21 promotes radiation-resistance of NSCLC [19], miR-125b regulates human NSCLC cell apoptosis via the phosphatidylinositol 3-kinase/AKT/glycogen synthase kinase 3*β* (PI3K/AKT/GSK3*β*) signaling pathway [20], and miR-9 regulates NSCLC cell invasion and migration by targeting eukaryotic translation initiation factor 5A2 (EIF5A2) [21]; in addition, miR-628-3p and miR-425-3p expression is significantly higher in early-stage lung cancer, and miR-532 expression is significantly lower than in the other types [15]. However, how miR-628-3p functions in lung cancer and which gene it targets are not known. Based on the TargetScan prediction, we selected nine genes with high degree of integration that can bind with miR-628-3p; among the three downregulated genes,* AKT1* has an important role in lung carcinogenesis, and depletion of* AKT1* has antiproliferative and antimigratory effects [22]. miR-9500 represses lung tumorigenesis and metastasis by targeting* AKT1* [23].* CASP3* is a previously unidentified target of miR-137 and plays an essential role in miR-137-mediated lung cancer progression [24]. HSP90 plays an essential role in maintaining cellular protein homeostasis by acting as a molecular chaperone to aid in the folding and intracellular trafficking of its protein clients [25]. The in silico prediction indicated that* HSP90* was a potential target of miR-628-3p, and it was significantly downregulated by miR-628-3p, so* HSP90* was selected for further study.

We found that siRNA silencing of* HSP90* or* HSP90* downregulation by miR-628-3p overexpression promoted A549 cell apoptosis and inhibited A549 cell migration. qRT-PCR and western blotting confirmed that miR-628-3p mimic transfection into A549 cells downregulated* HSP90* expression, as did* HSP90* siRNA transfection. In addition, the luciferase assay indicated that miR-628-3p may regulate* HSP90* expression by targeting its 3′ UTR.

## 5. Conclusion

In summary, our results indicate that miR-628-3p promotes A549 cell apoptosis and inhibits A549 cell migration by negatively regulating* HSP90*. Further studies should consider the relationship between miR-628-3p and* HSP90*, which may reveal a novel therapeutic strategy for lung cancer.

## Figures and Tables

**Table 1:** siRNAs against *HSP90* gene.

siRNA	Sequence	Target mRNA sequence
siRNA 1	F: 5′- GCCCUAAGAGACAACUCAAdTdT-3′	5′-GCCCTAAGAGACAACTCAA-3′
	R: 3′- dTdTCGGGAUUCUCUGUUGAGUU-5′	
siRNA 2	F: 5′- GGAACGUGAUAAAGAAGUAdTdT-3′	5′-GGAACGTGATAAAGAAGTA-3′
	R: 3′- dTdTCCUUGCACUAUUUCUUCAU-5′	
siRNA 3	F: 5′- GCACCAGAAUGAAGGAGAAdTdT-3′	5′-GCACCAGAATGAAGGAGAA-3′
	R: 3′- dTdTCGUGGUCUUACUUCCUCUU-5′	

## Data Availability

The data used to support the findings of this study are available from the corresponding author upon request.
